# PLiCat: decoding protein–lipid interactions by large language model

**DOI:** 10.1093/bib/bbaf665

**Published:** 2025-12-11

**Authors:** Feitong Dong, Jingrou Wu

**Affiliations:** Department of Chemical Biology, School of Life Sciences, Southern University of Science and Technology, Xueyuan Avenue, Nanshan District, Shenzhen, Guangdong 518055, China; Department of Computer Science and Engineering, Southern University of Science and Technology, Xueyuan Avenue, Nanshan District, Shenzhen, Guangdong 518055, China; Australian Artificial Intelligence Institute, Faculty of Engineering and Information Technology, University of Technology Sydney, 15 Broadway, NSW 2007, Australia

**Keywords:** protein–lipid interaction, protein language model, ESMC, BERT, lipid-binding sites

## Abstract

Protein–lipid interactions are essential for many cellular processes, as proteins associate with diverse lipid molecules to exert distinct functions. However, existing approaches are limited in discriminating among lipid categories, leaving a gap in our understanding of lipid-binding specificity. Recent advances in protein language models have opened new possibilities for discovering novel sequence insights. Here, we introduce PLiCat (Protein–Lipid interaction Categorization tool), a sequence-based framework designed to predict the categories of lipids that interact with proteins. PLiCat employs a hybrid deep learning architecture that integrates ESMC with BERT, enabling accurate and interpretable classification across eight major lipid categories. Through attribution analysis, PLiCat uncovers the sequence-encoded lipid-binding signatures and highlights residues contributing to binding specificity. Notably, PLiCat shows potential for identifying lipid-binding sites hidden in protein sequences. Furthermore, PLiCat can be used to assess the impact of pathogenic mutations on lipid-binding events. Collectively, PLiCat provides the first computational tool for lipid category prediction from protein sequence alone, offering valuable insights into lipid recognition mechanisms, and with promising applications in guiding rational protein design. The PLiCat source code and processed datasets are available at https://github.com/Noora68/PLiCat.

## Graphical Abstract

**Graphical Abstract:**
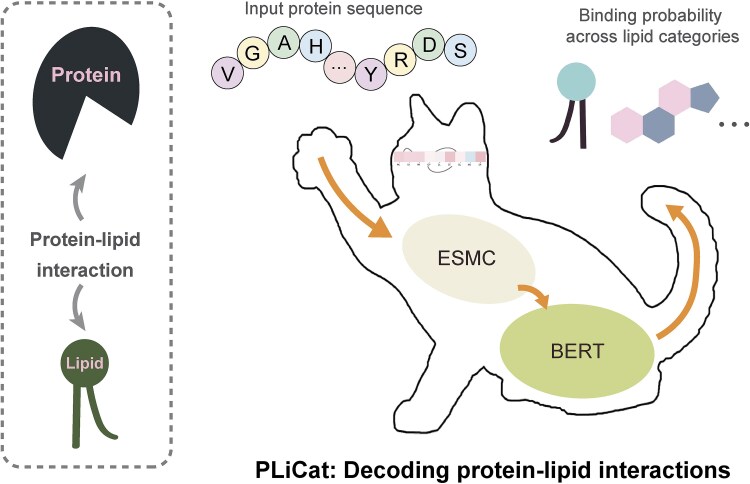

## Introduction

Lipids are a class of amphipathic biomolecules that play diverse roles in numerous cellular processes [1–3]. Beyond serving as structural components of membranes and energy reservoirs, lipids are increasingly involved in signal transduction, immune homeostasis, and epigenetic regulation [4–9], largely mediated through interactions with proteins [5, 9–11].

Many proteins selectively associate with specific lipid categories, and such lipid-binding specificity is a critical determinant of protein structure, function, and regulation [12]. According to LIPID MAPS, lipids are classified into eight major categories: Fatty Acyls (FA), Glycerolipids (GL), Glycerophospholipids (GP), Sphingolipids (SP), Sterol Lipids (ST), Prenol Lipids (PR), Saccharolipids (SL), and Polyketides (PK) (Supplementary Fig. 1) [13, 14]. Specific protein–lipid interactions can modulate protein structure and function in a variety of ways [15]. Therefore, understanding the patterns of protein–lipid interaction is essential for unraveling the intricate biological processes.

Traditional approaches for the identification of protein–lipid interactions rely on biochemical, biophysical, and structural methods, which are time-consuming and laborious [16]. Computational approaches based on known lipid-binding domains or amino acid properties are limited in discovering novel interactions [17]. The flexibility and complexity of protein–lipid interactions make it challenging to predict the preference of a certain protein for different lipid categories, highlighting the need for predictive tools in both research and protein design.

Recent advances in deep learning and protein language models (PLMs) offer opportunities to learn sequence–function relationships directly from amino acid sequences [18–20]. In particular, PLMs have demonstrated strong potential across a range of protein-related tasks, including structure prediction, antigen–antibody interaction, and subcellular localization [19, 21–28]. Despite these advances, it remains unclear whether PLMs can capture the hidden sequence features that govern protein–lipid binding.

To address the aforementioned issues, we present PLiCat (Protein–Lipid interaction Categorization tool), leveraging ESM Cambrian (ESMC) [21] and Bidirectional Encoder Representation from Transformers (BERT) modes [29] trained on a comprehensive lipid-binding protein dataset. PLiCat achieves superior performance in classifying the eight major lipid-binding categories, provides residue-level interpretability through attribution analysis, and evaluates the impact of pathogenic mutations. Overall, PLiCat enables prediction of lipid-binding specificity and decoding of sequence determinants underlying protein–lipid recognition. It further facilitates the understanding of lipid-associated protein functions and their therapeutic implications.

## Results

### PLiCat is a novel framework tailored for predicting protein–lipid interactions

To investigate the codes in protein sequences responsible for lipid binding, we set out to develop PLiCat, a predictive tool to distinguish sequence features of proteins binding to different lipid categories. It uses protein sequence as the only input, without requiring complex structural information or physicochemical properties.

**Figure 1:**
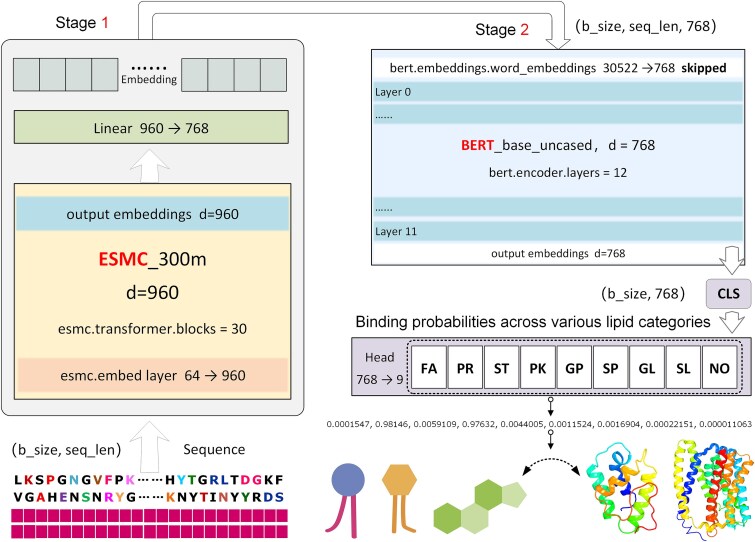
Overall schematic framework of PLiCat. The PLiCat model consists of two stages. In the first stage, the 300 M-parameter ESMC_300m model was trained to acquire high-quality embeddings from the input protein sequences. A linear layer (960, 768) was defined for dimensionality reduction. In the second stage, the BERT module takes the output embeddings from the ESMC module to learn relevant protein representations. Finally, the CLS vector from the output of the last transformer block of the BERT model was fed into a custom classification head to produce a multilabel classification vector. b_size: batch size. seq_len: sequence length.

As illustrated in Fig. 1, PLiCat integrates two components: the ESMC module, a large pretrained PLM, is optimized for efficiently extracting evolutionary-scale features [21]. In parallel, BERT uses bidirectional attention to capture long-range sequence relationships [29]. By combining ESMC with BERT, our architecture is designed to capture both evolutionary and long-range contextual dependencies, which are complementary representations for predictions.

The training and evaluation dataset is based on an open-source protein–lipid database known as BioDolphin [30]. The dataset encompasses a variety of samples, enabling comprehensive evaluation of lipid-binding patterns. About half of the data come from well-studied model organisms (Supplementary Fig. 2A), and mostly range from 100 to 400 amino acids (Supplementary Fig. 2B). Moreover, the lipid-binding proteins can be divided into four different types based on their membrane environment: integral membrane proteins, peripheral membrane proteins, soluble proteins, and lipid-anchored proteins [23]. The dataset is mostly soluble and transmembrane proteins, with fewer peripheral or lipid-anchored proteins, reflecting heterogeneous membrane associations (Supplementary Fig. 2C). Cross-distribution analysis shows most lipid categories are evenly represented across species and membrane types, with some context-specific enrichments (Supplementary Fig. 2E and 2F). The diversity of protein data enables the model to learn generalizable features across various species and membrane-association types.

After training, the Classification (CLS) token embedding from the final Transformer block of BERT, summarizing the entire sequence, is fed into a custom classification head, producing a nine-dimensional vector representing the multilabel predictions for the eight lipid categories plus a “none” class (Fig. 1).

### PLiCat accurately discriminates among lipid-binding categories

In a multilabel classification task, imbalanced data can substantially influence model performance (Supplementary Fig. 2D). To avoid overfitting and the long-tail effect, we conducted a 10-fold cross-validation on the training set. For each fold, the training and validation sets were divided in a 9:1 ratio (Table S1). Monitoring the training revealed consistent trends across metrics on both training and validation sets over epochs, suggesting the model learned stable and generalizable sequence representations without notable degradation, supporting the robustness of our training procedure (Supplementary Fig. 3). For rigorous evaluation, all 10 models were tested on an independent dataset. PLiCat consistently achieved high performance across Accuracy, Precision, Recall, F1 score, Area Under the Curve of the Receiver Operating Characteristic (AUC-ROC), and Area Under the Curve of the Precision–Recall (AUC-PR), with minor variations among folds (Supplementary Fig. 4), indicating good generalization and stability across different subsets of the data.

**Figure 2:**
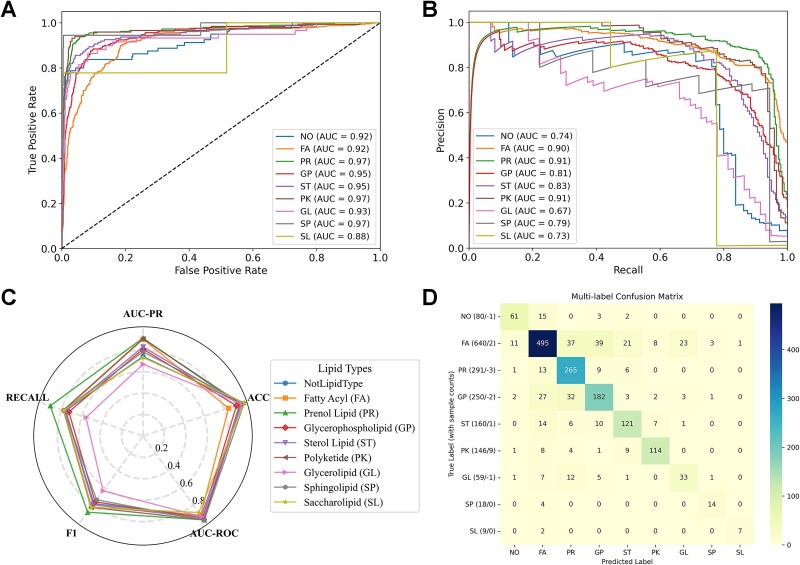
Model performance evaluation on the test dataset. (A) Receiver operating characteristic (ROC) curve with area under the curve (AUC) values. (B) Precision–recall (PR) curve with AUC values. (C) Radar chart summarizing multiple evaluation metrics. (D) Confusion matrix illustrating predictive performance across classes.

The final model was retrained on the entire training dataset, thereby leveraging all available data to maximize learning capacity. Performance was assessed across individual lipid categories to account for class imbalance (Fig. 2C, Table S4). PLiCat achieves excellent performance in AUC-ROC across all categories (0.88–0.97), reflecting high confidence in distinguishing true positives from negatives (Fig. 2A). Meanwhile, AUC-PR revealed varying performance across different lipid categories, which represents a precision–recall tradeoff, and is more sensitive to class imbalance. Among the eight lipid categories and the negative category, PR (0.91), FA (0.90), and PK (0.91) show the highest precision–recall performance. GP (0.81) and ST (0.83) show moderate performance in AUC-PR curves, while NO (0.74), GL (0.67), SP (0.79), and SL (0.73) displayed relatively lower precision–recall characteristics (Fig. 2B). Notably, even the lower-performing categories in AUC-PR (e.g. SL and GL) maintained high AUC-ROC values, suggesting the weaker precision–recall tradeoff is likely due to class imbalance (Fig. 2C).

The confusion matrix shows correctly predicted samples along the diagonal and misclassified samples on the off-diagonal, highlighting both category accuracy and the categories that are most frequently confused (Fig. 2D). FA, PR, and PK demonstrate strong performance, whereas GP and ST exhibit confusion with FA, and GL was occasionally misclassified as PR (Fig. 2D). These results suggest that the model has learned robust features and gained high confidence in the imbalanced dataset.

To benchmark PLiCat, we compared it with conventional machine-learning models [Random Forest (RF), Logistic Regression (LR), and Support Vector Machine (SVM)] and PLM-based models (ESM-2 [19], ProtBert [31]) on the same dataset. PLiCat consistently outperformed all baselines, demonstrating superior ability to capture lipid-recognition features (Supplementary Fig. 5 and Table S5).

### Ablation studies support the effectiveness of ESMC and BERT modules

To validate the contribution of different model components, we conducted ablation experiments by removing or modifying individual modules while keeping the rest unchanged. Several variant models were devised as follows: first, we removed the ESMC module, resulting in PLiCat-A. Second, we removed the BERT module, utilizing only the ESMC model for fine-tuning, generating PLiCat-B. Third, we froze all parameters of the pretrained ESMC module and fine-tuned only the BERT module, termed PLiCat-C. Fourth, we froze all parameters of ESMC and the first four layers of BERT, fine-tuning the last eight layers of BERT, termed PLiCat-D. Fifth, we fine-tuned the last six layers of ESMC and all layers of BERT while freezing the remaining layers, termed PLiCat-E (Supplementary Fig. 6). Each variant was trained under the same conditions as the PLiCat model. The performance of all the above ablation models is summarized in Table S6. As shown in Supplementary Fig. 6G, excluding the ESMC module resulted in a drop in sample accuracy and recall, indicating that ESMC plays a crucial role in capturing lipid-relevant sequence features. Consistently, omitting the BERT encoder and relying solely on the ESMC module led to a less severe effect in performance degradation, suggesting that evolutionary-scale features extracted by ESMC provide complementary information not captured by BERT alone. Similarly, freezing part of the model layers also results in a slight decline in model performance.

We further evaluated two factors affecting model performance: an unweighted loss variant (PLiCat-F) and a model with double negative samples (PLiCat-G). In both cases, PLiCat outperformed the variants, indicating that the weighted loss function and the original negative sample size are both reasonable and effective (Supplementary Fig. 6G and Supplementary Fig. 7, Table S6 and S7). These results collectively highlight that each module contribute indispensably to optimal performance.

### PLiCat learned interpretable representations of distinct lipid-binding categories

**Figure 3:**
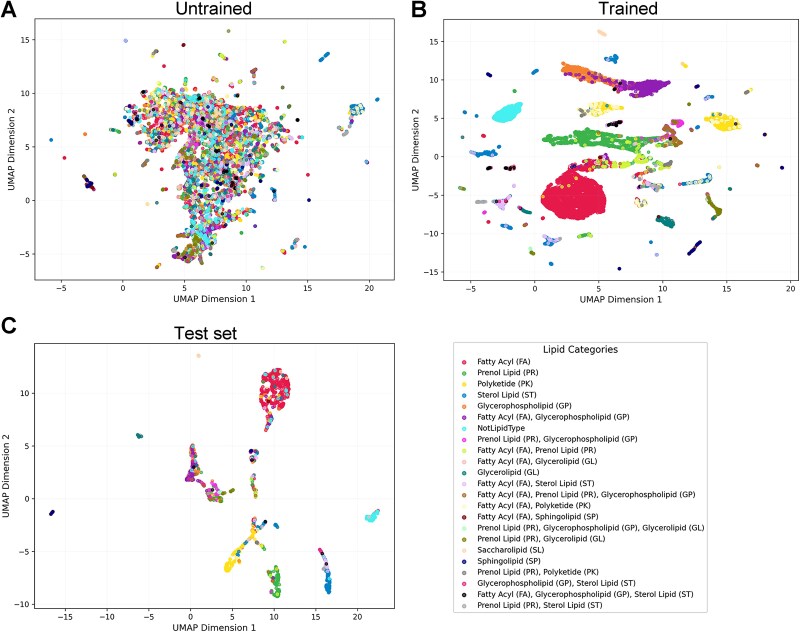
Visualization of the model’s latent space embeddings. (A) UMAP projection of sequence embedding distribution before training. (B) UMAP projection of sequence embedding distribution after training. (C) UMAP projection of sequence embedding distribution for the test set. Each point corresponds to a single data sample, with colors representing different lipid category labels.

Since PLiCat predicts the probability of association with different lipid categories, these probability distributions reflect the model’s view of lipid-binding preferences. To understand how PLiCat captures lipid-binding specificity, we examined the structure of the model’s latent space for interpretable representations. We extracted high-dimensional CLS embeddings for each protein sequence and projected them into a 2D space using UMAP [32]. The results reveal that the untrained model’s features are scattered, whereas the trained model shows aggregated structures with clear clustering into distinct regions (Fig. 3A and B). Cluster separation indicates the model can distinguish classes based on sequence-derived embeddings, while overlapping regions may reflect proteins with shared or ambiguous features (Supplementary Fig. 8). Moreover, the test set showed similar patterns, with well-separated clusters for each class, demonstrating robust generalization (Fig. 3C).

**Figure 4:**
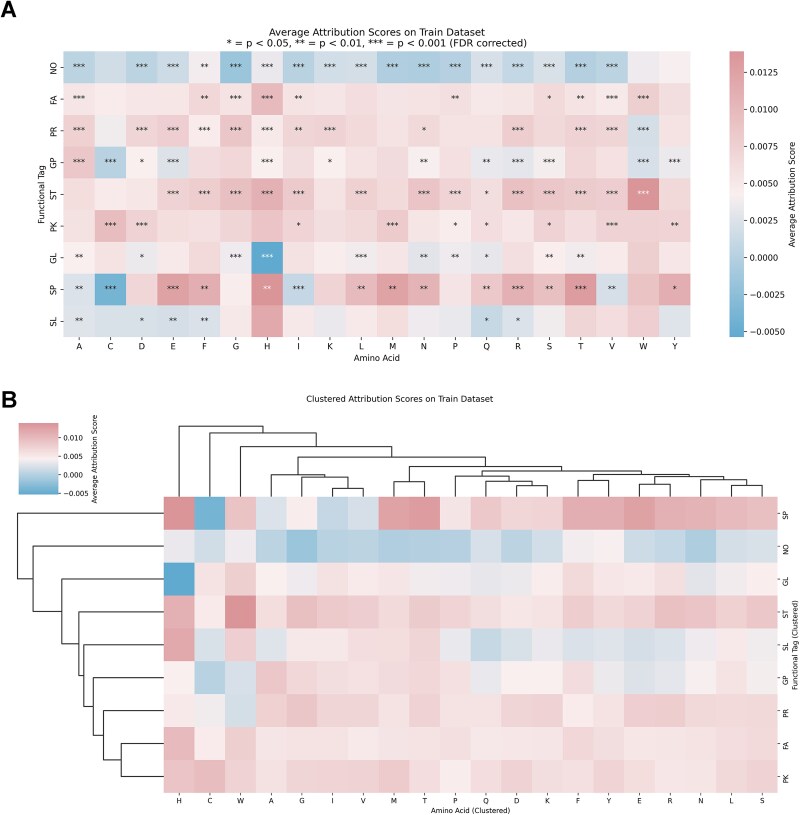
Attribution scores of amino acids for different lipid-binding categories predictions. (A) Heatmap visualization of attribution scores, illustrating the contribution of each amino acid to the prediction for different lipid categories. Rows correspond to amino acids. Columns represent eight lipid categories and negative category. (B) Clustering of different categories by attribution scores of amino acids.

To explore the biological basis of PLiCat predictions, we computed average attribution scores of each amino acid for each lipid category. Notably, the heat map shows that distinct lipid categories displayed differential binding propensities toward specific amino acid residues (Fig. 4A). For example, tryptophan (W) shows a positive contribution toward binding with ST and FA, but negatively to PR and GP. This pattern illustrates that each lipid category possesses a unique amino acid “signature”, reflecting selective interactions. Some lipid categories, such as SL, showed relatively weak or inconsistent signals across most residues, implying a reliance on global or contextual features rather than specific residue types (Fig. 4). Interestingly, several residues exhibit strong preference regardless of their hydrophobic nature, suggesting that additional factors, such as residue size, charge, polarity, or the local structural context, might play important roles in guiding lipid-specific recognition. This highlights the complexity of protein–lipid interactions and suggests that models capturing multidimensional residue features can provide deeper insights into lipid-binding. Together, these results demonstrate that the model’s latent space is not only high-capacity but also interpretable, highlighting the model’s ability to capture discriminative features from protein sequences.

### PLiCat facilitates the prediction of lipid-binding residues in two representative proteins

**Figure 5:**
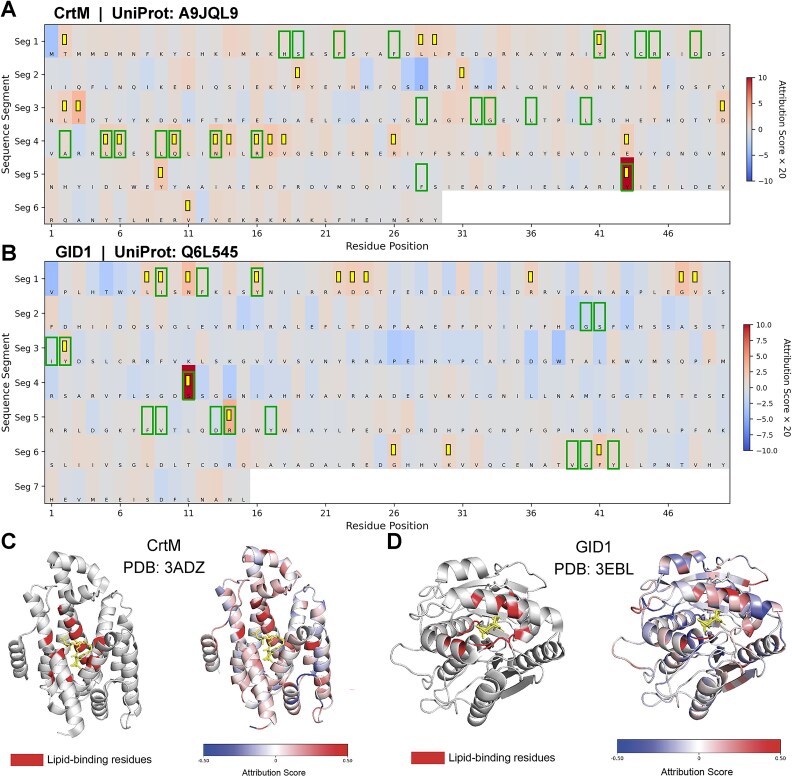
Identification of potential lipid-binding sites by amino acid contributions. (A, B) Heatmaps of attribution scores for CrtM and GID1. Heatmaps highlight residues with different attribution scores. Boxes indicate lipid-binding residues based on experimental validation. The blocks denote the same number of residues with the highest attribution scores outside the boxes. Overlaps between these boxes and blocks indicate correctly predicted lipid-binding residues based on the model’s attribution scores. Attribution scores range from negative (low) to positive (high), with the intensity of the visual representation reflecting the score strength. (C, D) Structural mapping of lipid-binding residues (left) and attribution scores (right) on protein 3D structures. Regions in the left panels correspond to experimentally determined lipid-binding residues. In the right panels, residues are colored according to their attribution scores (low to high, from least to most intense). Lipid molecules are shown as sticks. Comparison shows that residues with high attribution scores are spatially enriched around the lipid-binding sites.

We hypothesize that PLiCat’s predictions rely on sequence features enriched within or near lipid-binding pockets. These high-attribution regions likely correspond to residues involved in protein–lipid interactions, thereby allowing indirect inference of lipid-binding sites. To validate this, we applied the Integrated Gradients method to quantify the contribution of each input residue to the model’s prediction. By calculating attribution scores at the residue level, we identified sequence regions that are most influential for the outcome (Fig. 5).

We selected two lipid-binding proteins (UniProt IDs: A9JQL9 and Q6L545) for detailed analysis. Residues with top-ranked attribution scores (yellow blocks) partially overlap with actual binding residues (green boxes), indicating that the model can effectively identify lipid-binding regions (Fig. 5A and B).

A9JQL9, known as Dehydrosqualene synthase (CrtM), catalyzes the first committed step in staphyloxanthin biosynthesis in *Staphylococcus aureus*, mediating the condensation of two farnesyl diphosphate molecules [33–35]. Mapping attribution scores onto the CrtM structures in complex with Presqualene Diphosphate (PSPP), an unreactive substrate analog, demonstrates that residues with higher attribution scores cluster around experimentally determined lipid-binding sites (Fig. 5A and C).

The second protein, GID1 (GIBBERELLIN INSENSITIVE DWARF1), a gibberellin (GA) receptor in plants. GID1 specifically bind bioactive gibberellins, triggering conformational changes that facilitate downstream responses [36]. In the structure of GID1 bound to GA, the ligand occupies the pocket formed by the N-terminal lid and several loops. Notably, residues contributing most to the model’s prediction closely correspond to the known GA-binding sites, further supporting the model’s capability to identify true binding sites (Fig. 5B and D).

To quantitatively evaluate the attribution-based prediction, we compared the predicted lipid-binding sites with experimentally annotated sites from the processed BioDolphin dataset (6618 nonredundant sequences). When evaluated under a strict residue-level criterion (tolerance = 0), the F1 score was 0.10. Relaxing the tolerance to ±2 residues improved the score to 0.23, indicating that many predicted sites are spatially close to verified binding residues (Table S7). These results demonstrate the model’s potential to identify lipid-binding pockets and provide biologically plausible insights at the residue level.

### Effects of pathogenic mutations on lipid-binding events

Lipids play fundamental roles in many cellular processes, and dysregulation of lipid metabolism leads to various diseases [4, 5, 10, 11, 37]. One of the possible applications of PLiCat is to investigate whether lipid-related pathogenic mutations alter lipid-binding or selectivity. To verify its feasibility, we collected a lipid-related pathogenic dataset comprising mutant sequences. To assess whether wild-type (WT) and mutant (Mut) sequences occupy distinct regions in the model’s representation space, we compared the embeddings and prediction distributions of WT and mutant protein sequences. Principal component analysis (PCA) projection reveals that WT and mutant samples were largely overlapping in the embedding space, indicating that the model’s internal representations were broadly preserved. A small subset of points is more dispersed in the PCA plot, indicating a possible model-recognized deviation from the canonical sequence behavior (Supplementary Fig. 9A).

To further quantify the shift in prediction profiles, we computed Jensen–Shannon (JS) divergence over the predicted probability distribution for each lipid category, as a measure of similarity in the model’s predictions between WT and mutant sequences. Mutant sequences exhibited marginally higher values for most lipid categories, while SP and SL exhibited minimal changes, indicating the mutations slightly disrupt the model’s prediction. Consistently, Wasserstein distance analysis of model logits identified NO and FA as the most mutation-sensitive classes, while other categories exhibited minimal changes. This distributional metric captures the magnitude of change in the model’s output caused by sequence perturbation (Supplementary Fig. 9B and C). Although direct experimental validation is limited, the observed trends across disease-associated variants illustrate how pathogenic mutations broadly reshape lipid-binding probabilities, emphasizing the importance of coarse-grained approaches analyses in revealing biological insights.

## Discussion

The advancement of PLMs has shown tremendous success in a wide range of applications. By treating protein sequences as “sentences” of amino acid “words,” PLMs capture implicit sequence patterns that enable prediction and classification. Given the central role of protein–lipid interactions in cellular function, deciphering sequence codes underlying lipid binding is essential. In this study, we developed PLiCat, leveraging fine-tuned ESMC and BERT models to predict protein–lipid interaction potentials across lipid categories. By pinpointing residue-level determinants of protein–lipid binding, PLiCat offers insights that extend beyond category-level prediction and can guide targeted experiments to better understand the molecular mechanisms underlying lipid specificity.

While PLiCat demonstrates overall strong performance, precision–recall curves reveal disparities across lipid categories. Some lipid subclasses, such as GL and SL, are more challenging to distinguish. To explore potential biological factors underlying this difficulty, we analyzed the domains of proteins associated with different lipid categories (Supplementary Fig. 10). The results suggest that the reduced performance likely reflects the presence of shared protein domains across multiple lipid classes. Notably, the apparent enrichment of these domains may be partly driven by the low representation of these classes in the dataset, indicating that both shared domain patterns and limited sample size contribute to the difficulty in distinguishing them. Although data augmentation could increase the sample size for such classes, currently there is no biologically validated strategy for sequence-level augmentation of lipid-binding proteins, and naive perturbation may introduce distributional artifacts. Future improvements may include expanding the dataset, integrating lipid structural information, and applying advanced representation learning to better distinguish rare or similar lipid categories.

Our results indicate that lipid-binding codes in proteins likely serve dual roles: one for correct structural folding and another for binding to specific lipid categories. Over evolutionary timescales, constraints on physicochemical features have likely emerged not only to support protein folding, but also to enable specific lipid-binding.

There are several key questions pending. First, the interactions between proteins and lipids are not always associated with specific biological functions; in some cases, they may merely serve as structural components within the membrane or complex [1, 38]. Future studies should focus on elucidating the physiological significance of lipid–protein interactions. Second, PLiCat mainly focuses on eight major lipid categories [14]. Our model lacks the higher resolution to distinguish subclasses within each lipid category. For instance, chain length selectivity can directly influence membrane fluidity, signaling pathways, and protein localization [38]. While category-level predictions suffice for large-scale annotation and mechanistic hypotheses, future work could incorporate ligand-conditioned or pocket-level features to achieve molecular-resolution lipid recognition.

Third, the selectivity of lipid binding may drive proteins to localize to distinct membrane regions or even alter their intrinsic functions, highlighting the critical role of lipid composition in regulating protein behavior [38]. In the future, leveraging the underlying characteristics learned by the model could be capable to guide the rational design of lipid binders with specific preferences, which represents a valuable research direction.

Fourth, compared to other protein-ligand binding, protein–lipid interactions often involve multiple contact points, high flexibility, and membrane-dependent effects. Leveraging transfer learning from protein-ligand models could be a valuable future direction.

Taken together, this approach provides an innovative framework and deepens our understanding of protein–lipid interactions. Such efforts could not only serve as a powerful tool for designing proteins with tailored membrane interactions, but also open new avenues for drug development and show promising potential in developing therapeutic strategies for lipid-related diseases.

## Materials and methods

### Datasets

To train the PLiCat model, we collected a lipid-binding protein dataset from BioDolphin [30]. BioDolphin is a curated collection of 127 000 protein–lipid interaction information. We downloaded the BioDolphin dataset from the website (https://biodolphin.chemistry.gatech.edu/). For dataset construction, we extract lipid-binding protein sequences and the corresponding lipid categories. The sequences in BioDolphin possess a considerable degree of redundancy. To generate a high-quality dataset, we performed group-by and removed duplicates based on protein sequences. Furthermore, sequences containing illegal characters were replaced by the canonical sequence obtained from UniProt. After this data cleaning process, we collected a lipid-binding protein dataset, resulting in 16 112 proteins with lengths between 35 and 500 amino acids. Given the considerable imbalance between different lipid categories, we analyzed the distribution of samples across different lipid-binding categories in the dataset. Labels occurring less than 50 times were filtered out, and their corresponding samples were excluded from the dataset.

For negative samples, the nonlipid-binding proteins were collected from UniProt using stringent criteria, yielding 7150 negative samples with sequence length between 35 and 500 amino acids. To reduce redundancy, negative samples were clustered using the CD-HIT [39] algorithm with an identity threshold of 70%. Subsequently, a total of 800 sequences were randomly selected from the filtered negative samples to maintain relative balance across the dataset.

The final dataset contains 12 873 positive samples and 800 negative samples. To ensure clear separation between classes, we confirmed that the positive and negative samples are mutually exclusive.

For data splitting, 10% of the proteins were selected to produce the independent test dataset, which was used solely for testing. The remaining 90% dataset was used for 10-fold cross-validation. The final training and testing datasets contain 12 296 and 1377 sequences, respectively (Table S1).

### Model architecture, training, and evaluation

The PLiCat model consists of two major components: ESMC and BERT. The pretrained ESMC model contains ~300 million parameters and produces an embedding dimension of d_ESMC_ = 960. The ESMC model comprises 30 Transformer blocks that employ Rotary Position Embedding (RoPE) for efficient encoding of positional information. Within each Transformer block, the feed-forward network undergoes a dimensionality transformation of (960, 5120) → (2560, 960). The shape of the input embedding layer is (64, 960), where 64 corresponds to the amino acid vocabulary size defined by ESM. Next, the ESMC representation was passed to BERT. Due to the embedding dimension of BERT is 768, a linear transformation of size (960, 768) was defined to reduce the dimensionality. All transformer layers of ESMC and BERT were fine-tuned. The BERT CLS token embedding was fed into a classification head comprising a 768-unit fully connected layer with dropout (*P* = .1), producing logits for lipid type prediction.

We employed a training and evaluation strategy consisting of 10-fold cross-validation followed by final model selection and evaluation on a held-out test set. For model training, PLiCat was trained end-to-end with a batch size of 16 and evaluated using weighted binary cross-entropy loss with logits (BCEWithLogitsLoss). Optimization was performed using the AdamW algorithm with default parameters and an initial learning rate of 2e-5. More details about the model and training parameters are summarized in Tables S2 and S3. All models were implemented in PyTorch 2.7.

To address the imbalance between positive and negative samples in the training set, we adopted a weighted loss function that assigns higher weights to rare classes, thereby increasing their contribution to the training objective. The class-specific weight was calculated according to the following equation:

${weight}_c$ =$\displaystyle\frac{N}{K\cdot{n}_{c.}}$where ${weight}_c$ denotes the weight assigned to class c, *N* is the total number of samples in the training set, *K* is the total number of classes, and *n_c_* is the number of positive samples in class c.

Using a weighted loss function during training is appropriate for mitigating class imbalance; however, to objectively evaluate the model’s generalization performance, we used the unweighted loss and metrics on the validation set. This avoids artificially amplifying the influence of rare classes during evaluation. For model evaluation, the model performance was assessed using multiple metrics, including AUC-ROC, AUC-PR, F1 score, Accuracy, and Recall. Accuracy was computed at both the label level and sample level. We reported macro-averaged scores across labels unless otherwise specified. For each fold, the model was trained on 90% of the fold data and validated on the remaining 10%. This procedure produced 10 independently trained models, which were subsequently used to perform inference on the test dataset, enabling assessment of performance robustness and variability across folds.

To systematically evaluate the effectiveness and superiority of the PLiCat architecture, we conducted comparison experiments using two representative PLMs, ESM-2 [19] and ProtBert [31], as baselines. Specifically, the backbone of the PLiCat framework was replaced by either the ESM2_t12 or ProtBert pretrained model, while keeping all other network components, optimization strategies, and dataset partitions identical. Each model was fine-tuned end-to-end under the same hyperparameter settings.

### Traditional machine learning baselines using physicochemical features

To benchmark PLiCat against baseline approaches, we trained and evaluated three non-deep learning models (RF, SVM, and LR) on the same training/validation/test set. All models were implemented in SciKit-Learn (v1.7.1), with physicochemical features of each protein computed by the ProtPy package as input.

Protein-level feature vectors were computed using ProtPy (v1.2.1). The features encompass seven major categories of amino acid physicochemical properties: hydrophobicity, normalized van der Waals volume, polarity, polarizability, charge, secondary structure propensity, and solvent accessibility. For each property, ProtPy performs a Composition–Transition–Distribution (CTD) transformation to encode sequence-level statistics. Briefly, Composition (C) represents the fraction of amino acids belonging to each of the three predefined classes (e.g. polar/neutral/hydrophobic). Transition (T) captures the frequency of transitions between classes along the sequence. Distribution (D) describes the relative sequence positions corresponding to the 1%, 25%, 50%, 75%, and 100% occurrences of each class.

Each property thus yields 21 CTD features (3 for Composition, 3 for Transition, and 15 for Distribution). Combined across the seven property types, this results in 147 dimensions, to which 20 amino acid composition frequencies are appended, yielding a final 167-dimensional feature vector per protein. These physicochemical features were used as input to the traditional machine learning classifiers.

### Calculation of attribution scores

To interpret the contribution of each residue in the input protein sequence to the model’s prediction, we employed Integrated Gradients (IGs) as implemented in Captum (v0.8.0). IG computes the integral of gradients with respect to a baseline input, attributing importance scores to each amino acid based on its contribution to the model output. We used an all-unknow (<unk > token) sequence as the baseline input and approximated the path integral using 50 steps.

### Latent space investigation of PLiCat

To investigate the structure of learned representations, we extracted latent embeddings of protein sequences from the trained model. Latent space analysis was performed on both training and test sets. For each protein, the latent embeddings from the final encoder layer were extracted. To visualize the structure of these high-dimensional embeddings, the Uniform Manifold Approximation and Projection (UMAP) method is implemented using the umap-learn library (0.5.9.post2) with default hyperparameters. In each UMAP projection, individual protein is shown as point and colored by their lipid-binding categories annotation.

### Evaluation of lipid-binding site predictions

To quantitatively assess the performance of our lipid-binding site predictions, we used a set-based evaluation approach. Predicted and experimentally annotated binding residues were converted into integer sets, and true positives (TPs), false positives (FPs), and false negatives (FNs) were computed based on exact or tolerant matches within a specified ±tolerance in residue positions. The F1 score was then calculated from these counts.

### Pathogenic variants dataset construction

To apply PLiCat in pathogenic mutations, we first developed a lipid-related pathogenic protein dataset specifically tailored to analyze the pathogenic effects. The pathogenic mutations dataset was sourced from multiple databases. Variants linked to Mendelian diseases were obtained from the ClinVar Variant Database [40]. Various Cancer-associated studies were available through cBioPortal, including The Cancer Genome Atlas (TCGA) and Therapeutically Applicable Research to Generate Effective Treatments (TARGET) [41, 42]. Genomic coordinates of cancer variants originally based on the hg19 reference genome were converted to hg38 using Liftover [43]. To restrict our focus to lipid-related diseases, Ensembl VEP v114 was used for variant annotation [44]. Synonymous mutations, duplication mutations, intron/UTR/regulatory region mutations were excluded, and DNA variants that led to the same amino acid change were treated as equivalent.

### Computation of JS divergence and Wasserstein distance

Jensen–Shannon divergence and Wasserstein distance were computed to quantify the differences between model outputs for WT and mutant protein sequences. For the Jensen–Shannon divergence, the predicted probability distributions P (WT) and Q (mutant) for each label were first obtained from the model outputs. The divergence was calculated as follows:


$$ {\boldsymbol{D}}_{\boldsymbol{JS}}\left(\boldsymbol{P}\mid |\boldsymbol{Q}\right)=\frac{\mathbf{1}}{\mathbf{2}}{\boldsymbol{D}}_{\boldsymbol{KL}}\left(\boldsymbol{P}\mid |\boldsymbol{M}\right)+\frac{\mathbf{1}}{\mathbf{2}}{\boldsymbol{D}}_{\boldsymbol{KL}}\left(\boldsymbol{Q}\mid |\boldsymbol{M}\right) $$

where $\boldsymbol{M}=\frac{\mathbf{1}}{\mathbf{2}}\left(\boldsymbol{P}+\boldsymbol{Q}\right)$ is a mixture distribution of P and Q.

The Wasserstein distance (Earth Mover’s Distance) was calculated to measure the minimal “cost” of transforming one distribution into another. For each label, the model’s unnormalized outputs (logits) for the WT and mutant sets were treated as empirical distributions. The first-order Wasserstein distance *W (P, Q)* was computed as follows:


$$ \boldsymbol{W}\left(\boldsymbol{P},\boldsymbol{Q}\right)=\underset{\boldsymbol{\gamma} \boldsymbol{\in}\prod \left(\boldsymbol{P},\boldsymbol{Q}\right)}{\boldsymbol{\operatorname{inf}}}{\mathbb{E}}_{\left(\boldsymbol{x},\boldsymbol{y}\right)\sim \boldsymbol{\gamma}}\left[{\left\Vert \boldsymbol{x}-\boldsymbol{y}\right\Vert}_{\mathbf{1}}\right] $$

where P, Q are two distributions, $\prod \left(\boldsymbol{P},\boldsymbol{Q}\right)$ denotes the set of all possible joint distributions with marginals P and Q. $\left\Vert \boldsymbol{x}-\boldsymbol{y}\right\Vert$represents the transportation cost on the real number line.

### Principal component analysis projection analysis

PCA was applied to visualize and compare the distribution of model embeddings derived from WT and mutant protein sequences. For each sequence, the final-layer embedding vector from the trained model was extracted and standardized to have zero mean and unit variance across each feature dimension. PCA was performed using the scikit-learn implementation, with the first two principal components (PC1 and PC2) used for 2D visualization. The total explained variance was calculated as the cumulative proportion of variance captured by the selected components, reflecting the extent to which the original feature variance was preserved in the reduced-dimensional space. The scatter plots of PC1 versus PC2 were generated to examine clustering patterns and potential distributional shifts between WT and mutant embeddings.

**Key Points**

- PLiCat is a novel framework to predict lipid categories in protein–lipid interactions which leverages protein language model.
- PLiCat achieves superior performance in distinguishing across eight major lipid categories on test dataset.
- PLiCat offers an interpretable tool for understanding lipid-binding codes hidden in protein sequences.
- PLiCat can be used to discover lipid-binding sites and evaluate effects of pathogenic mutations on lipid-binding events.

## Supplementary Material

Supplementary_Figure_1_bbaf665

Supplementary_Figure_2_bbaf665

Supplementary_Figure_3_bbaf665

Supplementary_Figure_4_bbaf665

Supplementary_Figure_5_bbaf665

Supplementary_Figure_6_bbaf665

Supplementary_Figure_7_bbaf665

Supplementary_Figure_8_bbaf665

Supplementary_Figure_9_bbaf665

Supplementary_Figure_10_bbaf665

PLiCat_final_Supplementary_Information_bbaf665

## Data Availability

The datasets used for training and testing are available at https://github.com/Noora68/PLiCat/tree/main/process_data.

## Code availability

The model source code is available at https://github.com/Noora68/PLiCat. The trained PLiCat model is available at https://huggingface.co/Noora68/PLiCat-0.4B.
