# Supplementary figures and images for: PLiCat: decoding protein–lipid interactions by large language model
Source: Brief Bioinform. 2025 Dec 11;26(6):bbaf665. doi: 10.1093/bib/bbaf665 (PMC12696715; doi:10.1093/bib/bbaf665)

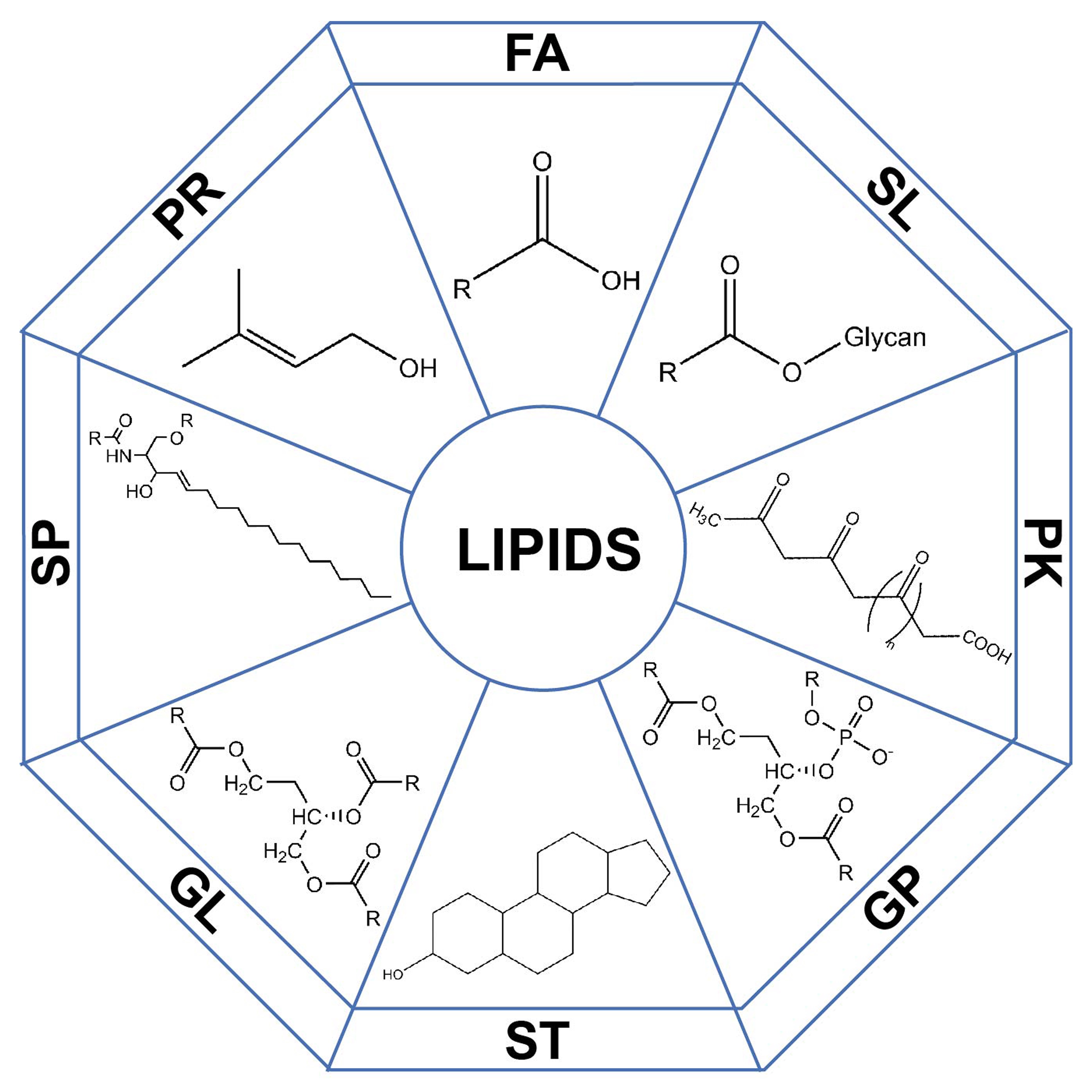

Supplement: Supplementary_Figure_1_bbaf665 [file supplementary_figure_1_bbaf665.jpeg]

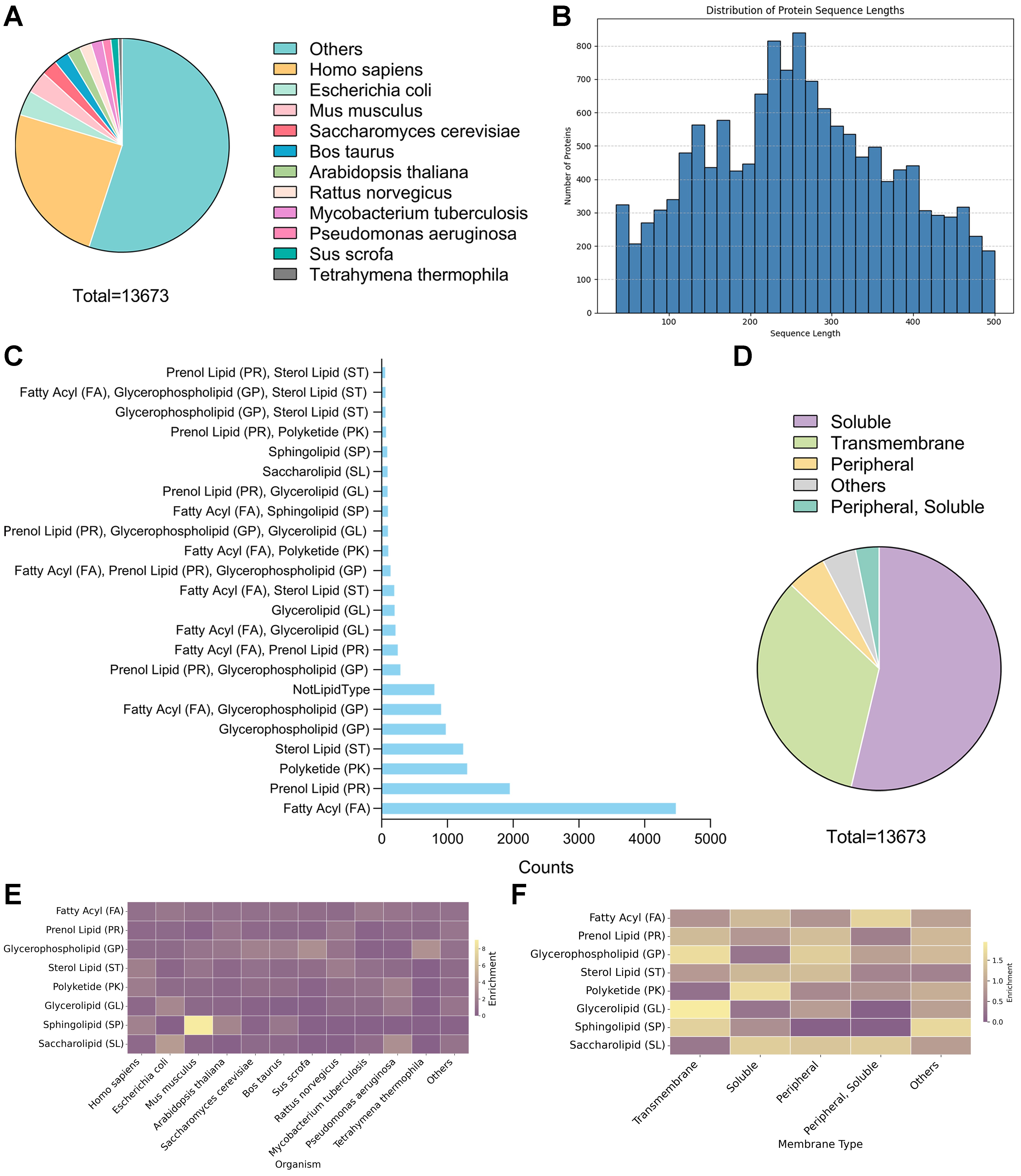

Supplement: Supplementary_Figure_2_bbaf665 [file supplementary_figure_2_bbaf665.jpeg]

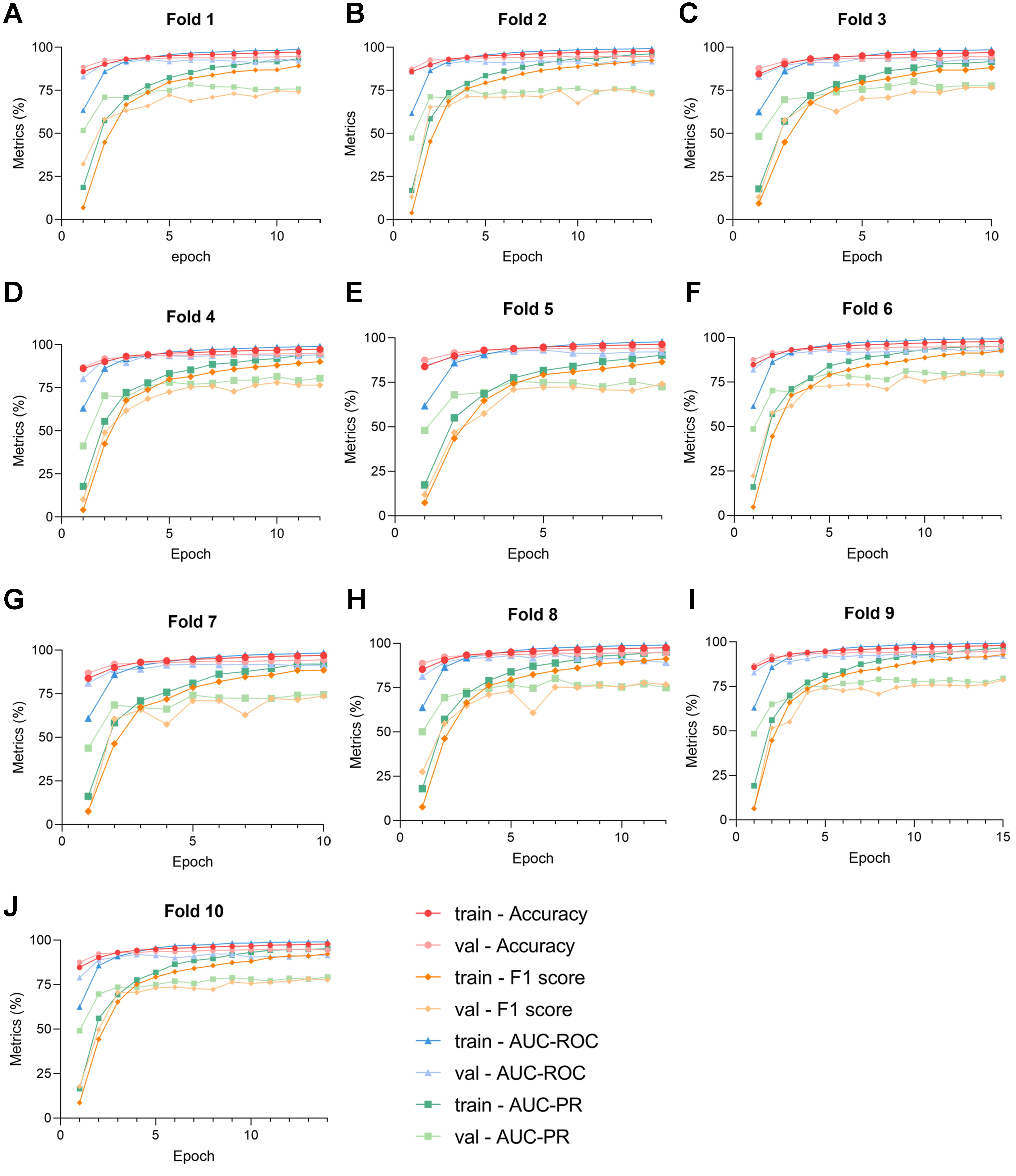

Supplement: Supplementary_Figure_3_bbaf665 [file supplementary_figure_3_bbaf665.jpeg]

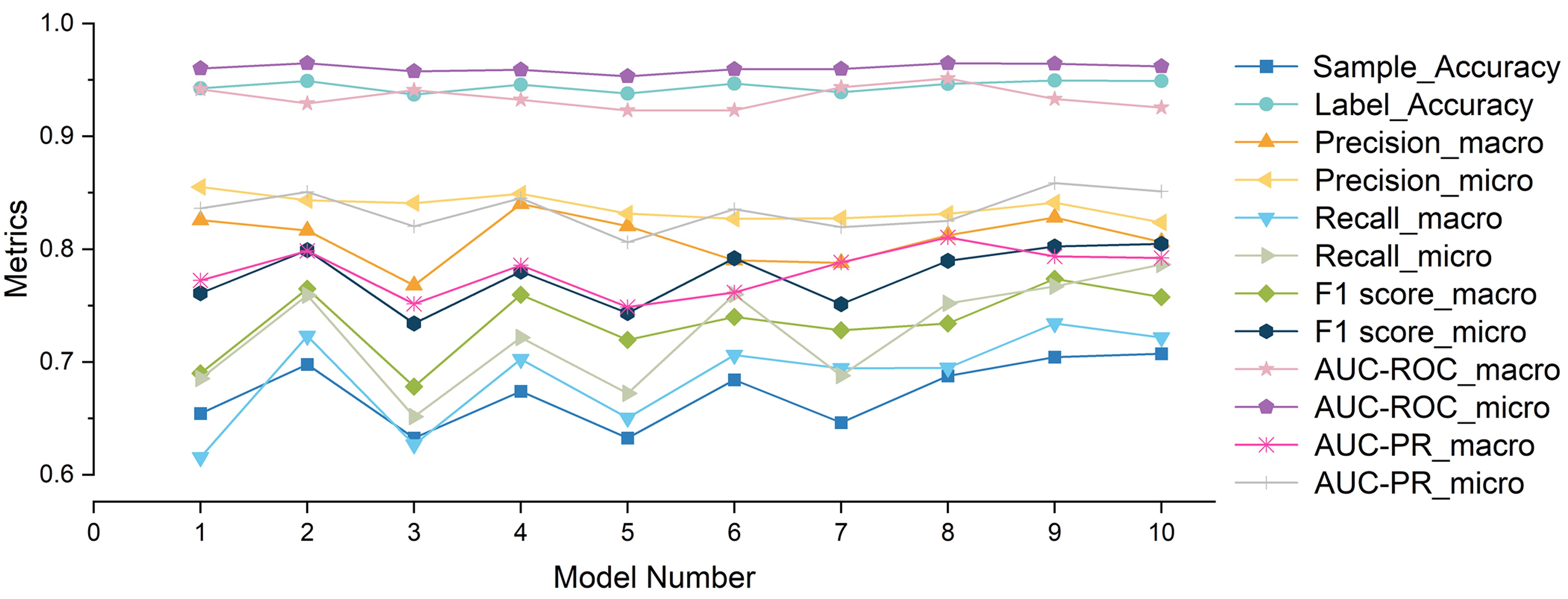

Supplement: Supplementary_Figure_4_bbaf665 [file supplementary_figure_4_bbaf665.jpeg]

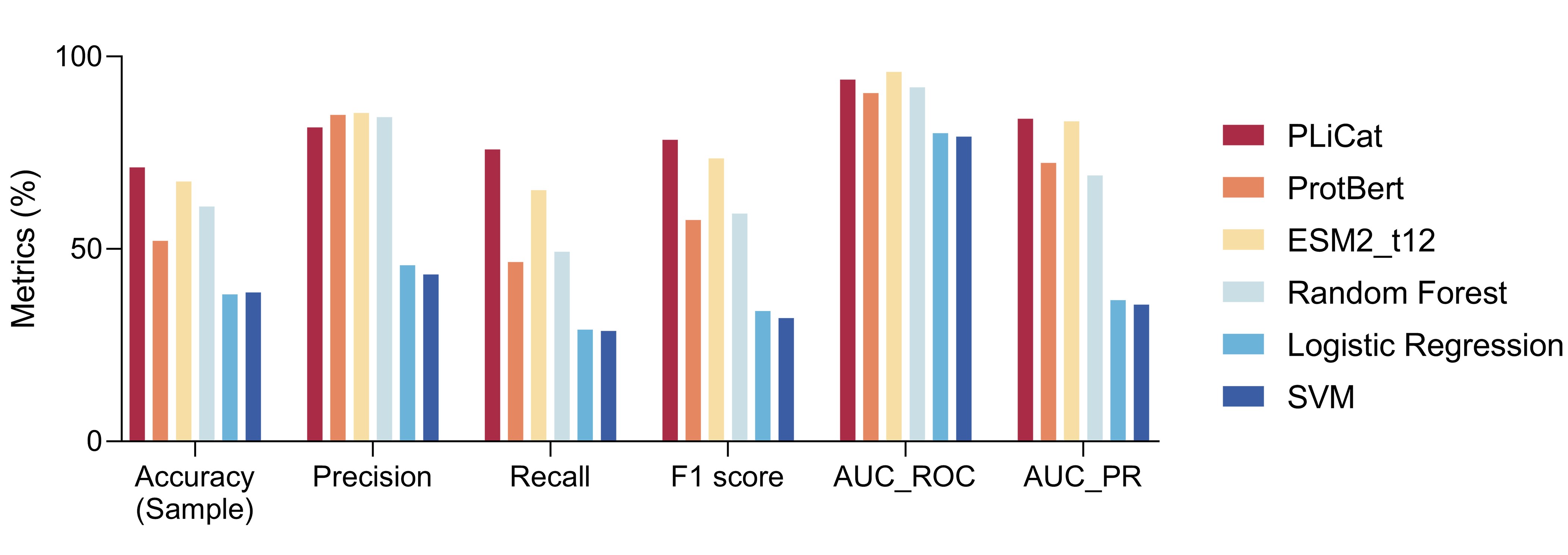

Supplement: Supplementary_Figure_5_bbaf665 [file supplementary_figure_5_bbaf665.jpeg]

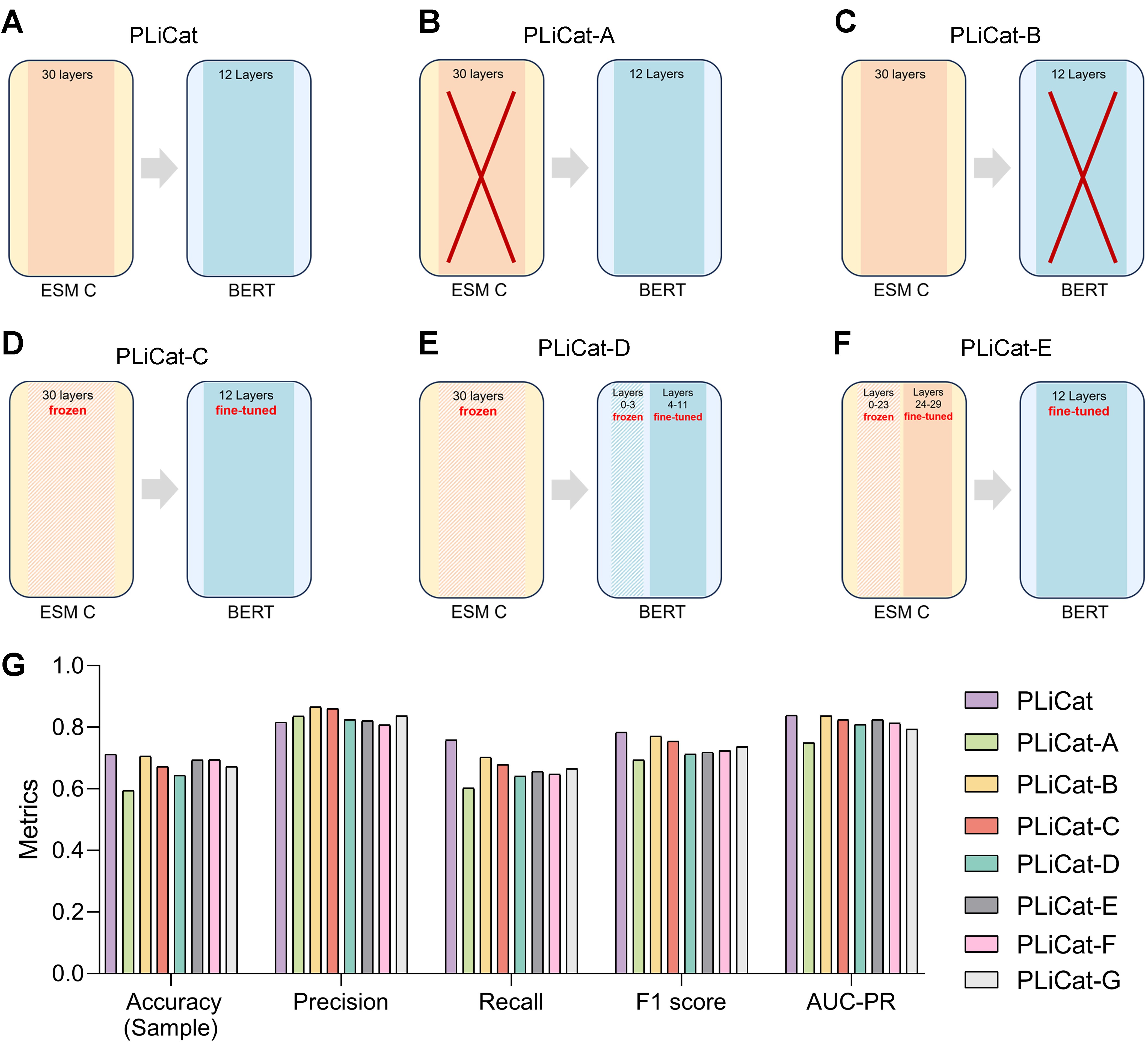

Supplement: Supplementary_Figure_6_bbaf665 [file supplementary_figure_6_bbaf665.jpeg]

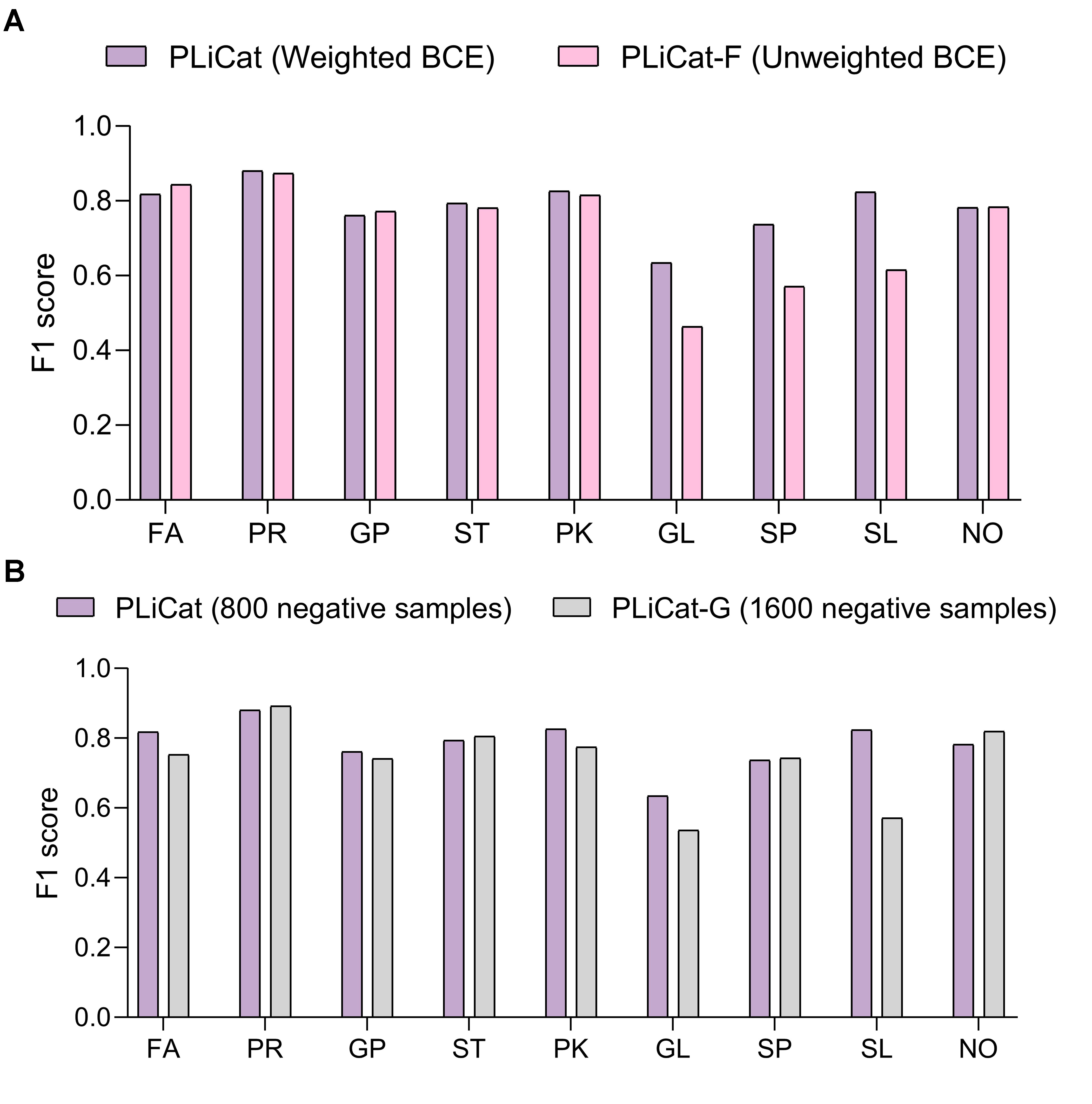

Supplement: Supplementary_Figure_7_bbaf665 [file supplementary_figure_7_bbaf665.jpeg]

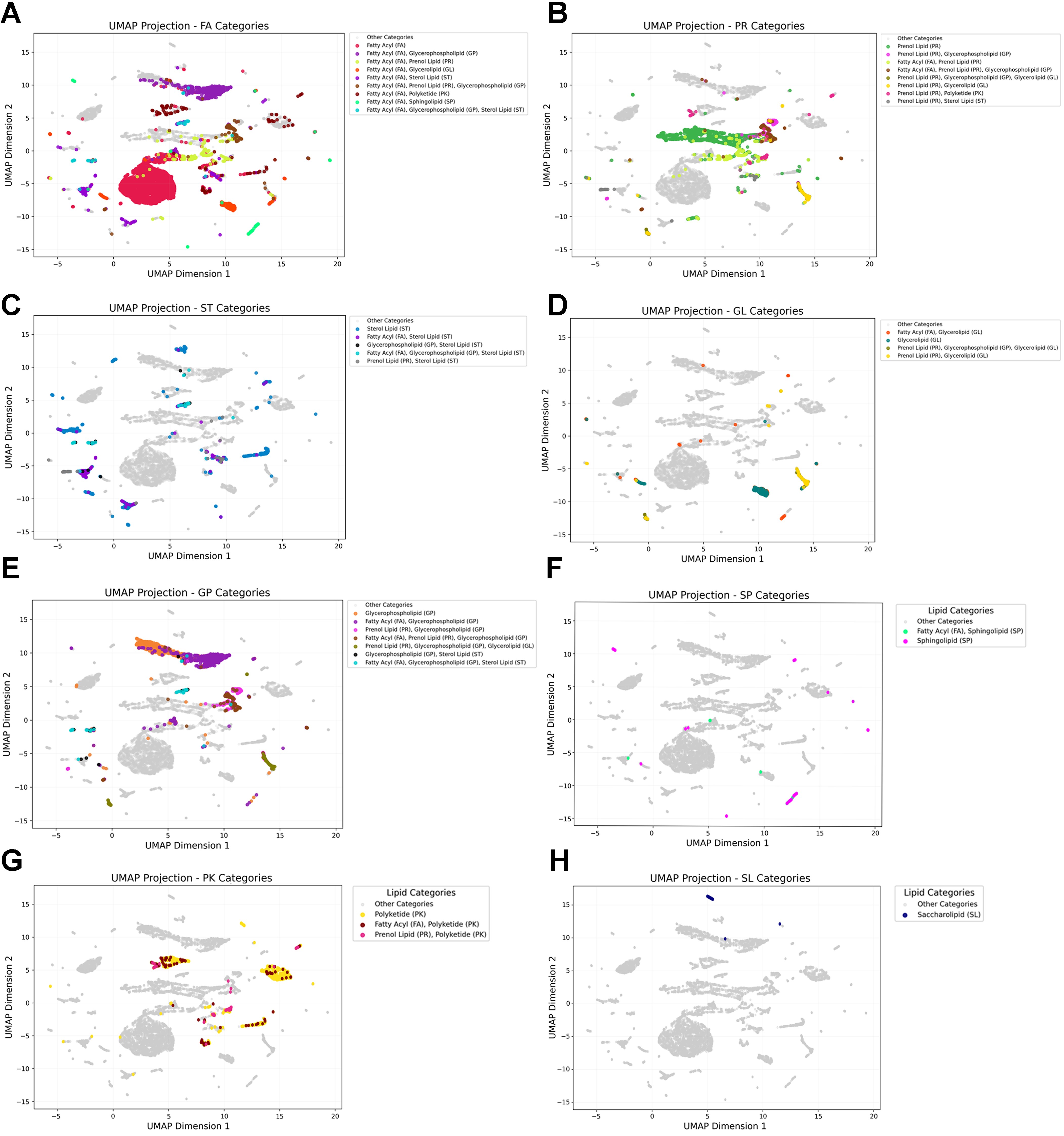

Supplement: Supplementary_Figure_8_bbaf665 [file supplementary_figure_8_bbaf665.jpeg]

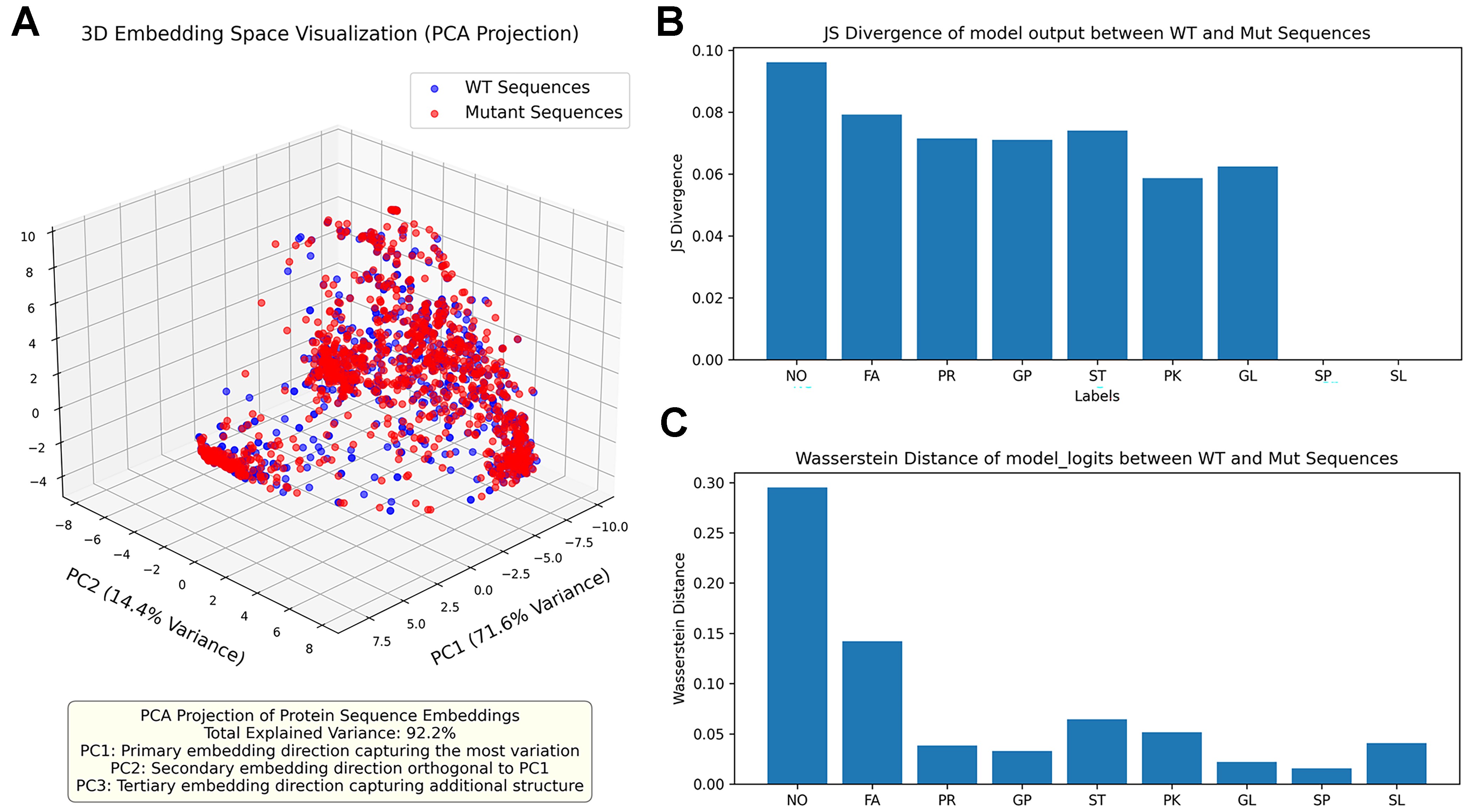

Supplement: Supplementary_Figure_9_bbaf665 [file supplementary_figure_9_bbaf665.jpeg]

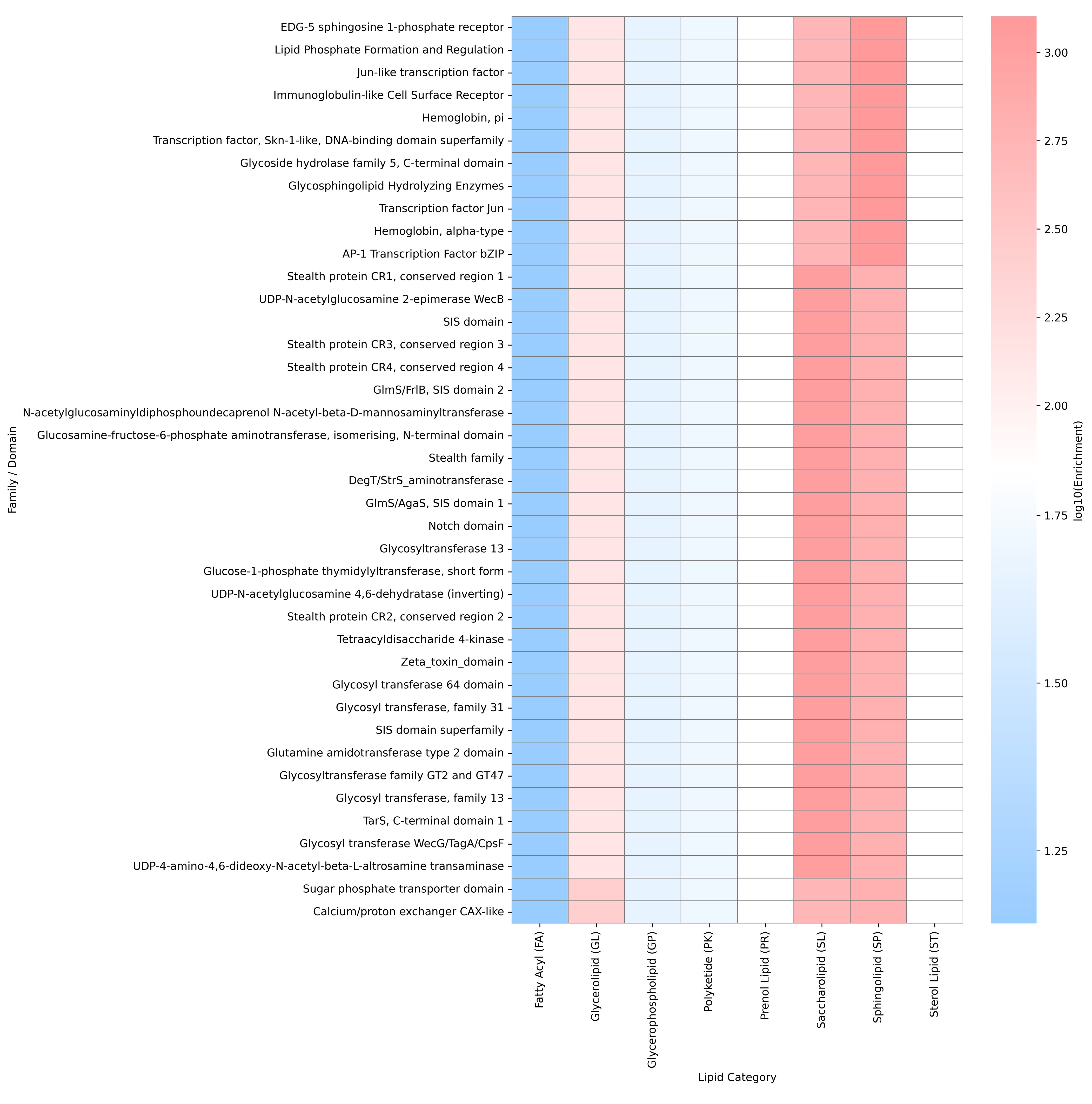

Supplement: Supplementary_Figure_10_bbaf665 [file supplementary_figure_10_bbaf665.jpeg]
