# Supplementary material for: PLiCat: decoding protein–lipid interactions by large language model
Source: Brief Bioinform. 2025 Dec 11;26(6):bbaf665. doi: 10.1093/bib/bbaf665 (PMC12696715; doi:10.1093/bib/bbaf665)
Supplement: PLiCat_final_Supplementary_Information_bbaf665 [file plicat_final_supplementary_information_bbaf665.docx]

**Supplementary Information**

**PLiCat: Decoding protein-lipid interactions by**

**large language model**

Feitong Dong^1,*^, Jingrou Wu^2,3^

^1^Department of Biology, School of Life Sciences, Southern University of Science and Technology, Shenzhen, Guangdong 518055, China.

^2^Department of Computer Science and Engineering, Southern University of Science and Technology, Shenzhen, Guangdong 518055, China.

^3^Australian Artificial Intelligence Institute, Faculty of Engineering and Information Technology, University of Technology Sydney, New South Wales 2007, Australia.

^*^To whom correspondence should be addressed: F.D. (12031011@mail.sustech.edu.cn)

**Supplementary Figure 1 Lipid classifications**

Graphical depiction of different lipid categories defined by LIPID MAPS. Fatty Acyls (FA), Glycerolipids (GL), Glycerophospholipids (GP), Sphingolipids (SP), Sterol lipids (ST), Prenol lipids (PR), Saccharolipids (SL), and Polyketides (PK).

**Supplementary Figure 2 Data distribution**

**(A)** Source organism distribution of the dataset, where major model species are shown individually and all other species are grouped under “Others”. **(B)** Statistical distribution of sequence lengths within the dataset. **(C)** Distribution of lipid-binding category labels. **(D)** Distribution of membrane-association types. Annotation of membrane association types was obtained from UniProt and supplemented with DeepLoc 2.1. **(E)** Heatmap showing the relative distribution of lipid categories across different species. The color scale indicates the magnitude of enrichment. **(F)** Heatmap depicting the relative distribution of lipid categories across different membrane-associated protein types. The color scale represents the magnitude of enrichment.

**Supplementary Figure 3 Monitoring of the metric trajectories of the 10-fold cross-validation**

For each fold, Accuracy, F1 score, AUC-ROC, and AUC-PR were recorded after each training epoch, illustrating the evolution of model performance over the training process. (A-J): Fold 1 - Fold 10. val: validation set.

**Supplementary Figure 4 Comparison of evaluation metrics for models using 10-fold cross-validation on the test dataset**

Each point represents the evaluation of one of the ten models trained during 10-fold cross-validation. Metrics are shown for both micro-averaged and macro-averaged calculations, including Accuracy (Sample and Label), Precision, Recall, F1 score, Area Under the Curve of the Receiver Operating Characteristic (AUC-ROC), and Area Under the Curve of the Precision-Recall (AUC-PR).

**Supplementary Figure 5 Performance comparison between PLiCat and baseline models**

Benchmark results of PLiCat against traditional machine learning classifiers (Random Forest, Logistic Regression, and SVM) and fine-tuned protein language models (ProtBert and ESM2_t12) on the same training and evaluation splits. Six evaluation metrics are reported: sample-level Accuracy, Precision, Recall, F1 score, AUC-ROC, and AUC-PR. PLiCat consistently achieves the highest or near-highest performance across all metrics, indicating improved performance compared to both conventional classifiers and other protein language models.

**Supplementary Figure 6 Ablation Study**

**(A-F)** Schematic diagrams of different ablation models. **(A)** The original PLiCat model architecture. **(B)** PLiCat-A: Removing the ESMC module, fine-tuning using only the BERT module. **(C)** PLiCat-B: Removing the BERT module, fine-tuning using only the ESMC module. **(D)** PLiCat-C: Using a pre-trained frozen ESMC model and fine-tuning the BERT model. **(E)** PLiCat-D: Freezing the ESMC module and the first 0-3 layers of the BERT module, fine-tuning the last 8 layers of the BERT module. **(F)** PLiCat-E: Freezing the first 24 layers of ESMC, fine-tuning the last 6 layers of ESMC, and the BERT module. **(G)** Comparative performance of different ablation models across multiple evaluation metrics. PLiCat-F adopts an unweighted binary cross-entropy loss. PLiCat-G doubled the number of negative samples.

**Supplementary Figure 7 Impact of loss function and negative sample size on the model performance across lipid categories**

(A) Performance of PLiCat trained with class-weighted BCE loss versus standard BCE loss (PLiCat-F), which accounts for label imbalance. (B) Performance of PLiCat trained with 800 negative samples versus 1600 negative samples (PLiCat-G).

**Supplementary Figure 8 UMAP projection of protein embeddings for different lipid categories**

Each subpanel (A-H) highlights a specific lipid category (colored dots) while other categories are shown in gray. **(A)** Fatty Acyl (FA, red), **(B)** Prenol Lipid (PR, green), **(C)** Sterol Lipid (ST, blue), **(D)** Glycerolipid (GL, cyan), **(E)** Glycerophospholipid (GP, orange), **(F)** Sphingolipid (SP, purple), **(G)** Polyketide (PK, yellow), and **(H)** Saccharolipid (SL, navy).

**Supplementary Figure 9 Effects of pathogenic mutations on lipid binding events**

**(A)** PCA projection of the embeddings from wild-type (WT) and mutant (Mutant) sequences. **(B)** Jensen-Shannon (JS) divergence computed between wild-type (WT) and mutant (Mutant) datasets for each category. **(C)** Wasserstein distance computed between wild-type (WT) and mutant (Mutant) datasets for each category.

**Supplementary Figure 10 Enriched protein families / domains across lipid categories**

Heatmap showing the top 40 protein domains with the highest enrichment for binding to lipid categories. Each cell represents the log10-transformed enrichment of a domain for a specific lipid class. Colors range from blue (low enrichment) through white (median) to red (high enrichment). Families / Domains are mapped from InterPro IDs to descriptive names for clarity.

**Table S1 Positive and negative data for training and testing**

| **Dataset** | **Number of data** | |
| --- | --- | --- |
|  | **Positive** | **Negative** |
| Training set | 11576 | 720 |
| Test set | 1297 | 80 |

See methods for more detailed information.

**Table S2 Architectural Hyperparameters of PLiCat**

| **Module** | **Parameter** | **Value** | **Description** |
| --- | --- | --- | --- |
| ESMC | Number of Transformer Blocks | 30 | Model depth |
|  | Hidden layer vector dimension | 960 | Model width |
|  | Input embedding dimension | (64, 960) | 64 corresponds to vocabulary size |
|  | ESMC FFN | (960, 5120) → (2560, 960) | Asymmetric two-layer linear module |
|  | Number of attention heads | 15 | Enhance the model’s representational capacity |
|  | Per-head dimension | 64 | Vector dimension divided by the number of attention heads |
|  | Activation function | SwiGLU | Improve training stability |
|  | Linear | (960, 768) | From ESMC to BERT |
| BERT | Number of BERT-Base Uncased Transformer Blocks | 12 | Model depth |
|  | Hidden layer vector dimension | 768 | Model width |
|  | Input embedding dimension | (30522, 768) | Replaced by dimensionally reduced (960, 768) |
|  | Number of attention heads | 12 | Enhance the model’s representational capacity |
|  | Per-head dimension | 64 | Vector dimension divided by the number of attention heads |
|  | FFN | (768, 3072) → (3072, 768) | Dimension: d→4d→d |
|  | Activation function | GELU | Continuously differentiable, avoiding the gradient discontinuity present in ReLU |
|  | BERT classification head | (768, 9) | Multi-label classification vector |
| Number of Parameters | Total parameters | 443,818,057 | Relate to model architecture |
|  | Trainable parameters | 443,818,057 | Relate to fine-tuning strategy |

**Table S3 Training Hyperparameters of PLiCat**

| **Parameter** | **Value** | **Description** |
| --- | --- | --- |
| Training strategy | 10-fold cross-validation | Evaluate the model’s generalization ability |
| Dataset split | Proportional to the label distribution | Preserve class distribution in subsets |
| Optimization algorithm | AdamW | $\beta_{1}=0.9,\beta_{2}=0.95$ |
| Loss Function | BCEWithLogitsLoss | Weighted computation of training loss |
| Weight decay | 0.05 | L2 regularization strength |
| Maximum sequence length | 500 | Context window |
| Early stopping strategy | Validation loss is not decreasing for five consecutive epochs | Identify the optimal model and avoid overfitting |
| Learning rate scheduling strategy | initial learning rate: 2e-5, with a warmup + cosine decay schedule | Warmup phase accounts for 10% of the total training steps to improve stability in the early stages, followed by a cosine decay phase over the remaining 90% to enhance generalization in the later stages. |
| Gradient clipping | Maximum gradient norm of 1.0 | Improve training stability |
| Threshold for multi-label binary classification | 0.6 | Sigmoid function to compute the logits of the model outputs. |

**Table S4 PLiCat performance metrics of different lipid categories on the test dataset**

| **Category** | **Accuracy** | **Precision** | **Recall** | **F1 score** | **AUC-ROC** | **AUC-PR** |
| --- | --- | --- | --- | --- | --- | --- |
| FA | 0.840232 | 0.868421 | 0.773438 | 0.818182 | 0.91558 | 0.904602 |
| PR | 0.947712 | 0.85209 | 0.910653 | 0.880399 | 0.968743 | 0.914436 |
| GP | 0.917211 | 0.798246 | 0.728 | 0.761506 | 0.945132 | 0.811365 |
| ST | 0.954248 | 0.834483 | 0.75625 | 0.793443 | 0.952558 | 0.833522 |
| PK | 0.965142 | 0.876923 | 0.780822 | 0.826087 | 0.967256 | 0.907716 |
| GL | 0.972404 | 0.733333 | 0.559322 | 0.634615 | 0.930429 | 0.670354 |
| SP | 0.992738 | 0.7 | 0.777778 | 0.736842 | 0.973878 | 0.785799 |
| SL | 0.997821 | 0.875 | 0.777778 | 0.823529 | 0.884747 | 0.732134 |
| NO | 0.975309 | 0.802632 | 0.7625 | 0.782051 | 0.923174 | 0.742193 |

**Table S5** Comparison of PLiCat with baseline models

| **Method** | **Sample Accuracy** | **Precision** | **Recall** | **F1 score** | **AUC-ROC** | **AUC-PR** |
| --- | --- | --- | --- | --- | --- | --- |
| **PLiCat** | **0.712** | 0.816 | **0.759** | **0.784** | 0.940 | **0.839** |
| ProtBert | 0.521 | 0.849 | 0.466 | 0.575 | 0.905 | 0.724 |
| ESM2_t12 | 0.675 | **0.854** | 0.653 | 0.735 | **0.960** | 0.832 |
| Random Forest | 0.610 | 0.843 | 0.493 | 0.592 | 0.920 | 0.691 |
| Logistic Regression | 0.382 | 0.458 | 0.290 | 0.339 | 0.801 | 0.367 |
| SVM | 0.387 | 0.434 | 0.287 | 0.320 | 0.792 | 0.355 |

**Table S6 Ablation Studies**

|  | **Sample accuracy** | **Label accuracy** | **Precision** | **Recall** | **F1 score** | **AUC-ROC** | **AUC-PR** |
| --- | --- | --- | --- | --- | --- | --- | --- |
| PLiCat | **0.712** | 0.951 | 0.816 | **0.759** | **0.784** | 0.940 | **0.839** |
| PLiCat-A | 0.595 | 0.936 | 0.836 | 0.603 | 0.694 | 0.930 | 0.750 |
| PLiCat-B | 0.706 | **0.954** | **0.867** | 0.703 | 0.771 | **0.971** | 0.837 |
| PLiCat-C | 0.671 | 0.948 | 0.860 | 0.678 | 0.754 | 0.962 | 0.824 |
| PLiCat-D | 0.644 | 0.944 | 0.824 | 0.640 | 0.712 | 0.957 | 0.808 |
| PLiCat-E | 0.693 | 0.949 | 0.821 | 0.656 | 0.719 | 0.965 | 0.824 |
| PLiCat**-**F | 0.695 | 0.943 | 0.808 | 0.648 | 0.724 | 0.927 | 0.814 |
| PLiCat-G | 0.672 | 0.947 | 0.837 | 0.665 | 0.737 | 0.943 | 0.794 |

PLiCat: employs a weighted binary cross-entropy loss - nn.BCEWithLogitsLoss(weight=train_class_weights)

PLiCat-F: adopts an unweighted binary cross-entropy loss - nn.BCEWithLogitsLoss()

PLiCat-G: Use 1600 negative samples

**Table S7 Quantitative evaluation of attribution-based lipid binding site prediction under different positional tolerances**

| **Tolerance** | **F1 score** |
| --- | --- |
| 0 | 0.1004 |
| ± 1 | 0.1834 |
| ± 2 | 0.2313 |
